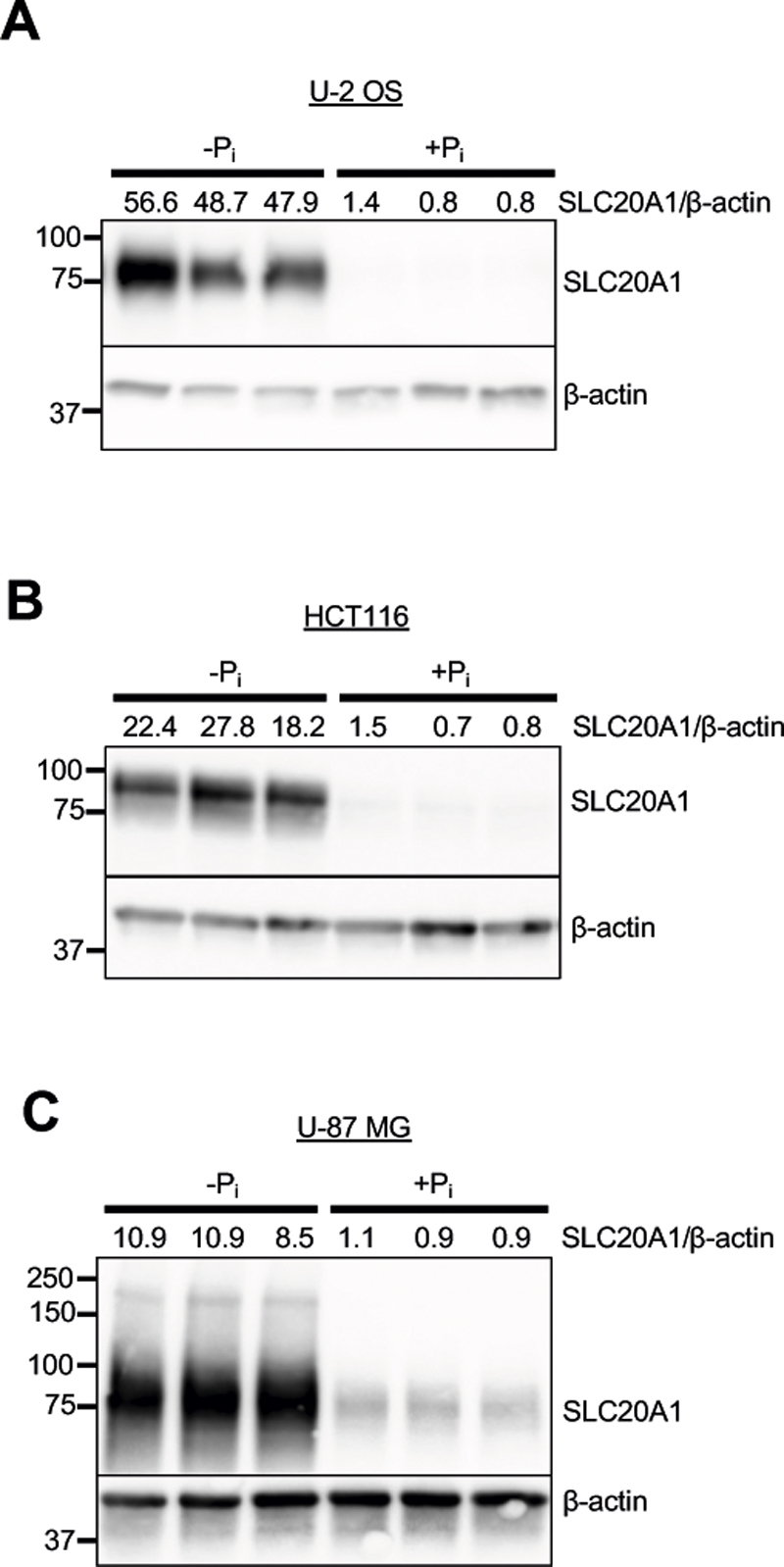# Cellular abundance of sodium phosphate cotransporter SLC20A1/PiT1 and phosphate uptake are controlled post-transcriptionally by ESCRT

**DOI:** 10.1016/j.jbc.2022.101945

**Published:** 2022-04-18

**Authors:** Christoph Zechner, W. Mike Henne, Adwait A. Sathe, Chao Xing, Genaro Hernandez, Shengyi Sun, Mi Cheong Cheong

**Affiliations:** 1 Division of Endocrinology, Department of Internal Medicine, University of Texas Southwestern Medical Center, Dallas, Texas, USA; 2 Charles and Jane Pak Center for Mineral Metabolism and Clinical Research, University of Texas Southwestern Medical Center, Dallas, Texas, USA; 3 Department of Pharmacology, University of Texas Southwestern Medical Center, Dallas, Texas, USA; 4 Department of Cell Biology, University of Texas Southwestern Medical Center, Dallas, Texas, USA; 5 Eugene McDermott Center for Human Growth and Development, University of Texas Southwestern Medical Center, Dallas, Texas, USA; 6 Department of Bioinformatics, University of Texas Southwestern Medical Center, Dallas, Texas, USA; 7 Department of Population and Data Sciences, University of Texas Southwestern Medical Center, Dallas, Texas, USA; 8 Department of Molecular Biology, University of Texas Southwestern Medical Center, Dallas, Texas, USA; 9 Center for Molecular Medicine and Genetics, Wayne State University School of Medicine, Detroit, Michigan, USA

**Keywords:** membrane transport, cell surface protein, protein degradation, endosomal sorting complexes required for transport, cell metabolism, CRISPR/Cas9, phosphate transporter, genome-wide forward genetic screen, ATCC, American Type Culture Collection, Cas9, CRISPR-associated protein 9, CHMP, charged multivesicular body protein, DAPI, 4′,6-diamidino-2-phenylindole, DMEM, Dulbecco's modified Eagle's medium, EGFP, enhanced GFP, ESCRT, endosomal sorting complexes required for transport, HEK293T, human embryonic kidney 293T cell line, sg, single guide, VPS, vacuolar protein sorting–associated protein

## Abstract

Inorganic phosphate is essential for human life. The widely expressed mammalian sodium/phosphate cotransporter SLC20A1/PiT1 mediates phosphate uptake into most cell types; however, while SLC20A1 is required for development, and elevated SLC20A1 expression is associated with vascular calcification and aggressive tumor growth, the mechanisms regulating SLC20A1 protein abundance are unknown. Here, we found that SLC20A1 protein expression is low in phosphate-replete cultured cells but is strikingly induced following phosphate starvation, whereas mRNA expression is high in phosphate-replete cells and only mildly increased by phosphate starvation. To identify regulators of SLC20A1 protein levels, we performed a genome-wide CRISPR-based loss-of-function genetic screen in phosphate-replete cells using SLC20A1 protein induction as readout. Our screen revealed that endosomal sorting complexes required for transport (ESCRT) machinery was essential for proper SLC20A1 protein downregulation in phosphate-replete cells. We show that SLC20A1 colocalizes with ESCRT and that ESCRT deficiency increases SLC20A1 protein and phosphate uptake into cells. We also found numerous additional candidate regulators of mammalian phosphate homeostasis, including genes modifying protein ubiquitination and the Krebs cycle and oxidative phosphorylation pathways. Many of these targets have not been previously implicated in this process. We present here a model in which SLC20A1 protein abundance and phosphate uptake are tonically negatively regulated post-transcriptionally in phosphate-replete cells through direct ESCRT-mediated SLC20A1 degradation. Moreover, our screening results provide a comprehensive resource for future studies to elucidate the mechanisms governing cellular phosphate homeostasis. We conclude that genome-wide CRISPR-based genetic screening is a powerful tool to discover proteins and pathways relevant to physiological processes.

Phosphate is indispensable for many biological functions, including DNA and RNA synthesis, energy storage, regulation of protein function, formation of plasma membrane lipids, proton buffering, and skeleton formation (1, 2, 3, 4). While phosphate homeostasis in humans is tightly regulated, many of the underlying molecular mechanisms are still poorly understood. Phosphate deficiency causes severe diseases, including rhabdomyolysis, seizures, and osteomalacia (5). Phosphate excess is associated with cardiovascular disease in the general population with apparent good health and in patients with chronic kidney disease, and vascular calcification has been identified as a main driver of morbidity and mortality (6, 7).

All cells require uptake of phosphate, which is mediated by the Na^+^-coupled inorganic phosphate transporter SLC20A1/PiT1. For a cellular process of such fundamental significance, our knowledge of the regulation of SLC20A1 activity in health and disease is surprisingly limited. Increased transcripts of *SLC20A1* have been reported in calcifying vascular tissue, which has been proposed to be pathogenic for soft tissue calcification (8, 9). Increased *SLC20A1* mRNA has been detected in 208F rat fibroblasts following phosphate starvation (10) and in aggressive malignant tumors (11, 12), presumably to restore cellular phosphate and accommodate rapid growth, respectively.

SLC20A1 is a 12 transmembrane-spanning cell surface protein that was initially identified as a retrovirus receptor and is now recognized to mediate uptake of phosphate into cells driven by the inward-directed sodium gradient and negative interior voltage (10, 13, 14, 15, 16). *SLC20A1* is expressed in a wide range of tissues, including brain, kidney, liver, lung, heart, and bone (10, 15, 17, 18), and its loss during embryogenesis is lethal (19). In addition to its role in mediating phosphate uptake, SLC20A1 has been postulated to have a transport-independent function for cell proliferation and implicated in extracellular sensing of phosphate concentrations (20, 21).

In the present study, we imposed the biologic stress of phosphate deprivation on cells to evoke the appropriate compensatory increase in phosphate uptake via SLC20A1. We found that SLC20A1 protein abundance was dramatically induced. However, *SLC20A1* mRNA was high at baseline, and its induction was only mild, implying a major contribution of post-transcriptional mechanisms in regulating SLC20A1 protein abundance and phosphate uptake in response to phosphate starvation. We propose that the low baseline SLC20A1 protein levels despite the abundant mRNA are due to a tonic negative suppression of SLC20A1 protein in phosphate-replete cells where unchecked phosphate entry would be detrimental. We capitalized on this biologic response and performed a genome-wide CRISPR/CRISPR-associated protein 9 (Cas9)–based loss-of-function genetic screen and found that SLC20A1 protein destruction through the endosomal sorting complexes required for transport (ESCRT) is a major post-transcriptional negative regulator of SLC20A1 protein levels that limits phosphate uptake into cells. Our screen furthermore unveiled an array of genes and pathways that have thus far not been linked to ESCRT machinery and phosphate homeostasis that are candidate regulators of SLC20A1 and phosphate homeostasis.

## Results

### SLC20A1 protein levels are markedly induced following phosphate starvation

**Figure 1:**
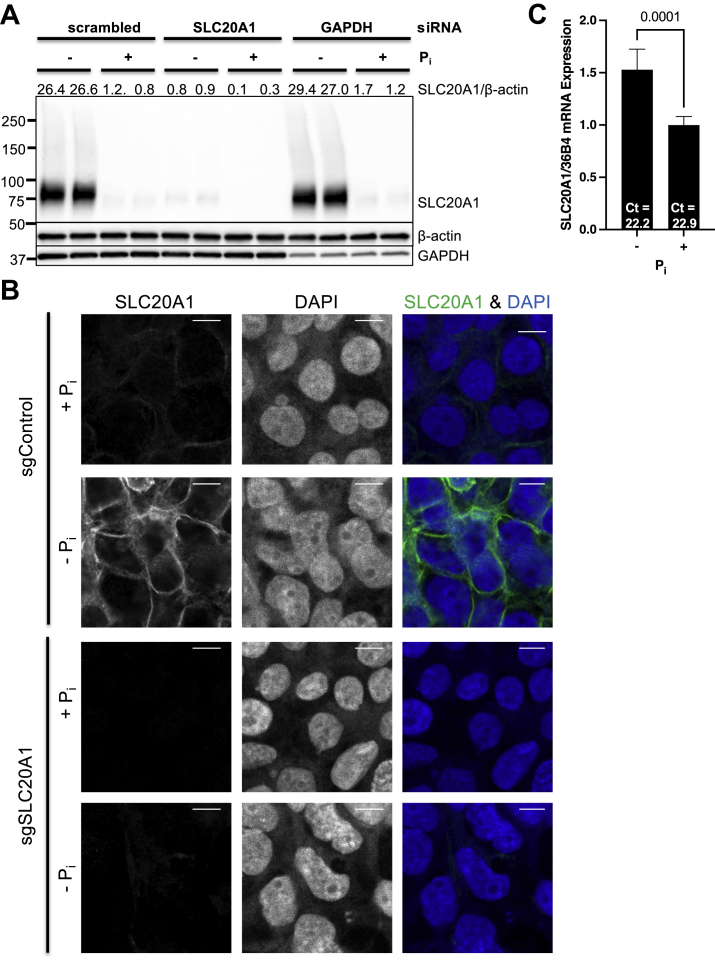
**Phosphate-starvation results in massive induction of SLC20A1 protein abundance in HEK293T cells.** Cells were phosphate starved (−Pi) for 48 h with phosphate-replete controls (+Pi). *A*, HEK293T cells were subjected to immunoblot analysis for SLC20A1 including siRNA-based knockdown for antibody validation (*top lane*) with β-actin loading control (*middle lane*). *GAPDH* siRNA was used as positive control for knockdown efficiency (*bottom lane*) and scrambled siRNA as negative control. Representative data of two experiments are shown. SLC20A1/β-actin abundance was determined by densitometry and normalized to the scrambled +Pi group (*top*). *B*, representative SLC20A1 immunofluorescence images (*left*) from sgControl-HEK293T (*top*) and sgSLC20A1-HEK293T cells (*bottom*) with DAPI as nuclear marker (*center*) and overlayed images on the *right* (SLC20A1 [*green*] and DAPI [*blue*]). The scale bars represent 10 μm. Representative data of three experiments are shown. *C*, *SLC20A1* mRNA expression was normalized to 36B4. n = 6. Bars represent mean ± SD. Ct, average cycle threshold; DAPI, 4′,6-diamidino-2-phenylindole; HEK293T, human embryonic kidney 293T cell line.

**Supplemental Figure S1:**
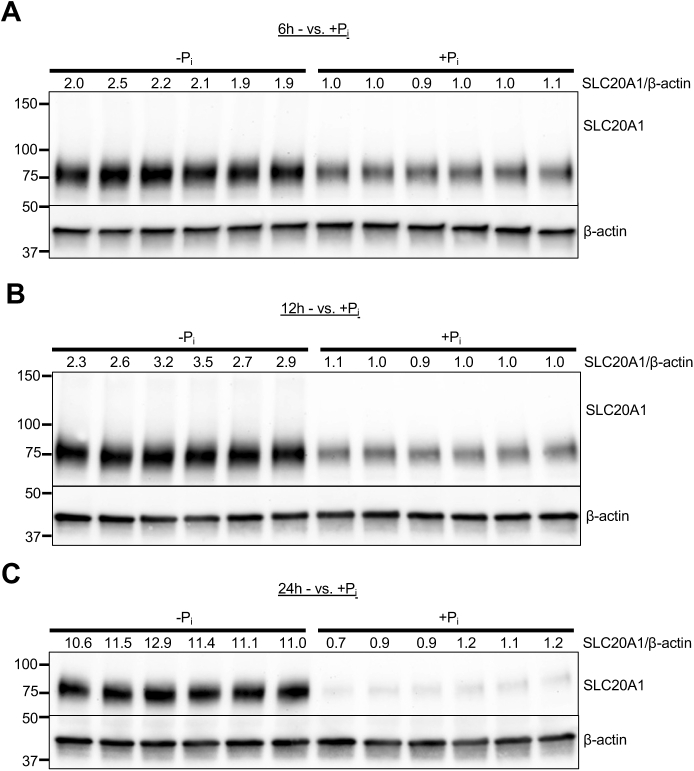


In order to obtain a readout to interrogate mammalian phosphate homeostasis, we examined native SLC20A1 protein abundance following phosphate starvation in human embryonic kidney 293T (HEK293T) cells. Immunoblot analyses revealed a progressive increase of SLC20A1 protein levels culminating in striking induction at the 24 and 48 h time points (Figs. S1, *A*–*C* and 1*A*). Knockdown of SLC20A1 confirmed the specificity of the SLC20A1 antibody that was used. Similarly, striking SLC20A1 protein induction was also observed in other cell lines originating from a variety of tissues including bone tumor–derived U-2 OS cells, colon cancer–derived HCT116 cells, and presumably glioblastoma-derived U-87 MG cells (Fig. S2, *A*–*C*), indicating that this phenomenon is well conserved and thus biologically relevant. Subsequent experiments were performed in HEK293T cells as this cell line demonstrates robust SLC20A1 induction following phosphate starvation, is easily maintained in culture, and is readily amenable to the molecular biological manipulations that were used for this project. As expected, immunofluorescent staining for SLC20A1 followed by confocal imaging in single-guide (sg)Control-HEK293T cells that were phosphate starved for 48 h confirmed the immunoblot findings and revealed SLC20A1 localization predominantly at the plasma membrane (Fig. 1*B*, *top*). Specificity of the utilized SLC20A1 antibody was verified using sgSLC20A1-HEK293T cells (Fig. 1*B*, *bottom*). Interestingly, phosphate starvation resulted in only a mild increase of already abundant *SLC20A1* mRNA levels (Fig. 1*C*), suggesting the presence of post-transcriptional mechanisms for SLC20A1 induction following phosphate starvation.

### Genome-wide loss-of-function genetic screen reveals ESCRT machinery as major limiter of SLC20A1 protein abundance

**Figure 2:**
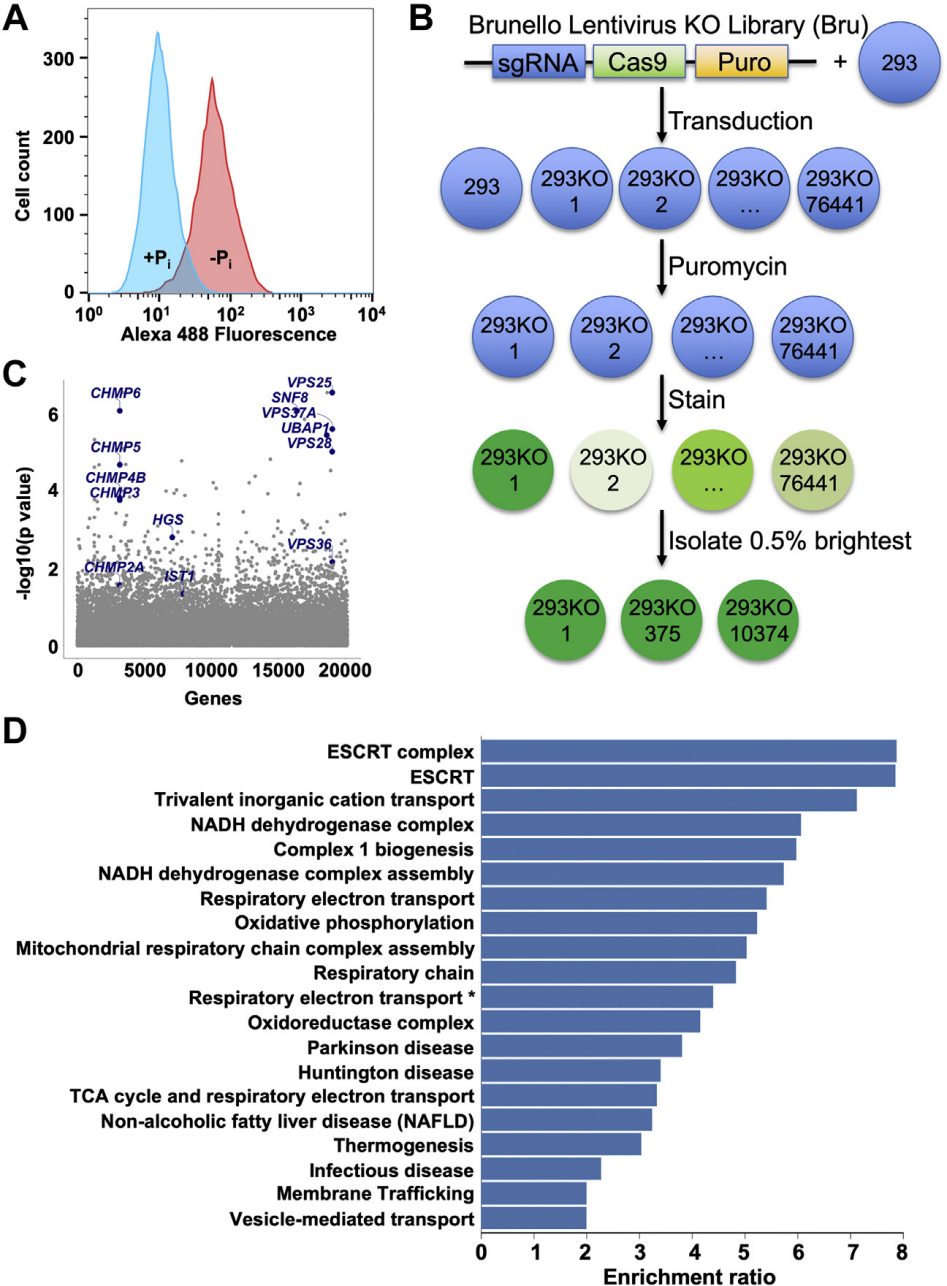
**Genome-wide loss-of-function genetic screen identifies endosomal sorting complexes required for transport (ESCRT) as major negative regulator of SLC20A1 protein levels.***A*, flow cytometry–based analysis of SLC20A1 protein abundance in phosphate-replete (+Pi) and 48 h phosphate-starved (−Pi) HEK293T cells. *B*, workflow of CRISPR/Cas9-based genetic screen. Shade of *green* of immunostained cells denotes fluorescence level with darker cells displaying higher fluorescence (=higher SLC20A1 protein levels). *C*, Manhattan plot of genetic screening results highlights loss-of-function guides of ESCRT subunits that are significantly enriched in the 0.5% brightest cells. *D*, WebGestalt-based analysis of genetic screening results displays pathways with enriched loss-of-function guides in the 0.5% brightest cells. False discovery rate was <0.05 for all displayed pathways. ∗Full pathway name is “Respiratory electron transport, ATP synthesis by chemiosmotic coupling, and heat production by uncoupling proteins.” Cas9, CRISPR-associated protein 9; DAPI, 4′,6-diamidino-2-phenylindole; HEK293T, human embryonic kidney 293T cell; Puro, puromycin resistance cassette; sgRNA, single-guide RNA.

Prior studies showed that SLC20A1 protein levels can be increased through activating transcription factor 4 and CCAAT/enhancer binding protein β–dependent transcription (9). In contrast, the discrepancy between the relatively weak mRNA but strong SLC20A1 protein upregulation following phosphate starvation suggests the dominant use of post-transcriptional mechanisms. To interrogate regulators of SLC20A1 protein levels, we employed flow cytometry using an SLC20A1 antibody and fluorescent secondary antibody to detect phosphate-dependent changes in SLC20A1 abundance. Phosphate starvation for 48 h resulted in a several-fold increase in SLC20A1 fluorescence (Fig. 2*A*), confirming that cellular SLC20A1 levels react in a physiologically appropriate fashion and can be surveyed in a high-throughput flow cytometry format that is suitable for large-scale genetic screening. Based on this finding, we performed a whole-genome CRISPR/Cas9-based loss-of-function genetic screen in phosphate-replete HEK293T cells to identify genes whose absence leads to increased SLC20A1 protein levels, that is, genes serving as basal negative regulators of SLC20A1 protein abundance (Fig. 2*B*). Bioinformatic analyses of the genetic screening results revealed that cells expressing KO guide RNAs targeting ESCRT proteins contributed many of the candidates that are significantly enriched in the top 0.5% brightest cells (Fig. 2*C*), and a majority of core ESCRT genes were significantly enriched in the 0.5% brightest cells (Table 1). The core ESCRT subunits VPS37 (vacuolar protein sorting–associated protein 37), CHMP2 (charged multivesicular body protein 2), and CHMP4 exist in several isoforms (22), and our screen provided evidence that only one isoform of each is involved in SLC20A1 degradation (VPS37A, CHMP2A, and CHMP4B, respectively; Table 1). Further pathway enrichment analyses confirmed that the most enriched pathway in the 0.5% brightest cells is in fact the ESCRT pathway (Fig. 2*D*). Taken together, these results suggest that ESCRT pathway proteins are important negative regulators of SLC20A1 protein abundance.

**Table 1:** Enrichment of ESCRT subunits in the 0.5% brightest screened cells

ESCRT complex	Gene name	*p*
0	***HGS***	0.0014858
	*STAM*	0.054817
	*STAM2*	0.68162
1 (Core)	***VPS37A***	0.00000222
	*VPS37B*	0.9717
	*VPS37C*	0.062909
	*VPS37D*	0.99895
	*TSG101*	0.11475
	***VPS28***	0.00000861
1 (Auxiliary)	***UBAP1***	0.0000032
	*MVB12A*	0.71259
	*MVB12B*	0.6581
2	***SNF8***	0.000000738
	***VPS25***	0.000000246
	***VPS36***	0.0064856
3 (Core)	***CHMP2A***	0.026348
	*CHMP2B*	0.73305
	***CHMP3***	0.00015776
	*CHMP4A*	0.61807
	***CHMP4B***	0.00013807
	*CHMP4C*	0.91839
	***CHMP6***	0.000000738
3 (Auxiliary)	*CHMP1A*	0.62986
	*CHMP1B*	0.57928
	***CHMP5***	0.000019
	*CHMP7*	0.66296
	***IST1***	0.044269
VPS4	*VPS4A*	0.51728
	*VPS4B*	0.053702
	*VTA1*	0.14614
Accessory	*PDCP6IP = ALIX*	0.052392
	***PTPN23***	0.00010805

Gene names in bold are significantly enriched (*p* < 0.05).

### Targeted loss-of-function experiments confirm that ESCRT machinery regulates SLC20A1 protein abundance

**Figure 3:**
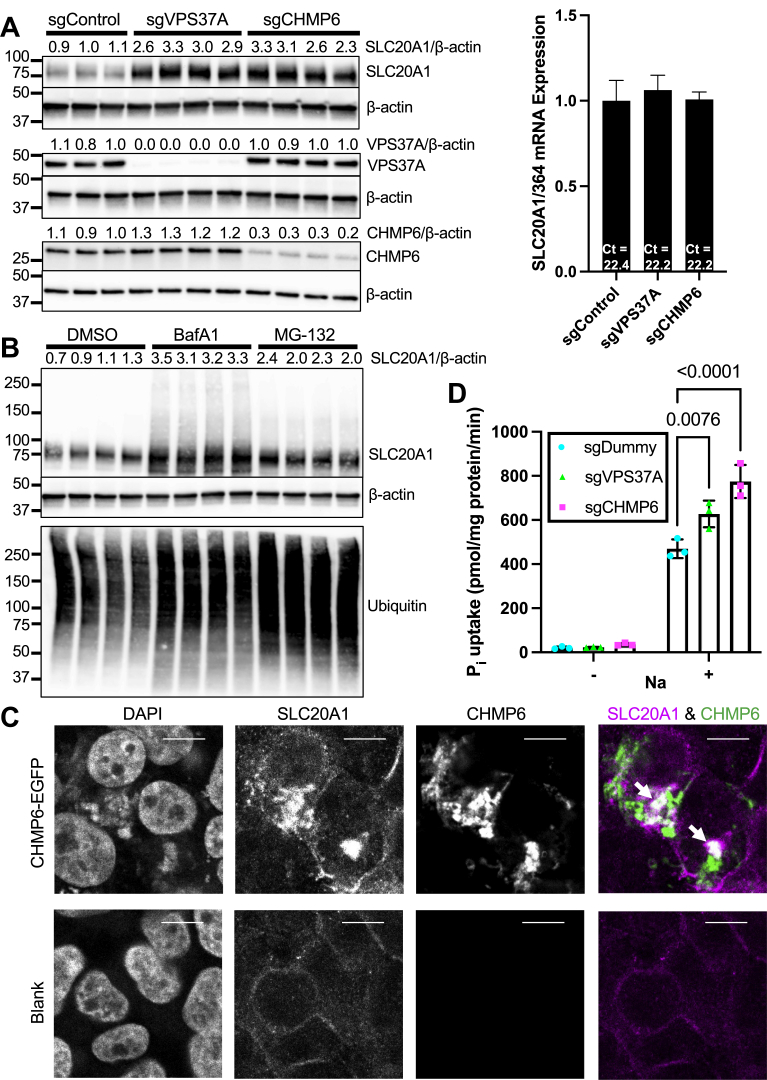
**Genetic and chemical inhibition of ESCRT/lysosomal protein degradation pathway confirm its role as direct negative regulator of SLC20A1 protein levels.***A*, *left*, immunoblot of SLC20A1 protein levels in sgVPS37A-HEK293T and sgCHMP6-HEK293T cells with sgControl (*top panel*). Immunoblots of VPS37A (*middle panel*) and CHMP6 (*bottom panel*) were performed for assessment of gene targeting efficiency. β-actin was used as loading control. Representative data of two experiments are shown. SLC20A1/β-actin abundance was determined by densitometry and normalized to the control group (*top*). *Right*, *SLC20A1* mRNA expression of sgVPS37A and sgCHMP6 cells with sgControl cells was normalized to *36B4*. n = 4. Bars represent mean ± SD. *B*, SLC20A1 immunoblot in HEK293T cells treated with lysosome inhibitor bafilomycin A1 (BafA1) or proteasome inhibitor MG-132 *versus* DMSO control for 24 h (*top panel*). β-actin was used as loading control (*middle panel*). SLC20A1/β-actin abundance was determined by densitometry and normalized to the DMSO group (*top*). Efficiency of BafA1 and MG-132 treatment was assessed using ubiquitin immunoblot, which was exposed for optimized visualization of smaller proteins (*bottom panel*). *C*, HEK293T cells were transfected with CHMP6-EGFP expression plasmid for 24 h (*green* in overlayed image) to induce arrest of ESCRT-mediated protein trafficking. Cells were then stained with SLC20A1 antibody and Alexa 594–coupled secondary antibody (*magenta* in overlayed image) and subjected to confocal imaging. DAPI was used as nuclear marker. *White arrows* denote colocalization between SLC20A1 and CHMP6-EGFP. Presented data are representative images of three experiments. The scale bars represent 10 μm. *D*, phosphate uptake in sgVPS37A and sgCHMP6 HEK293T cells was measured with sgControl cells as control (n = 3). Bars represent mean ± SD. Presented results are representative of two independent experiments. Ct, average cycle threshold; DAPI, 4′,6-diamidino-2-phenylindole; DMSO, dimethyl sulfoxide; EGFP, enhanced GFP; ESCRT, endosomal sorting complexes required for transport; HEK293T, human embryonic kidney 293T cell line; sg, single guide.

For validation of our genetic screening results, we created HEK293T cells deficient in the top-ranking proximal ESCRT I subunit *VPS37A* and the top-ranking distal ESCRT-III subunit *CHMP6*. Immunoblots confirmed complete loss of VPS37A in sgVPS37A cells (Fig. 3*A* *left graph*; *middle panel*), whereas 28% of CHMP6 immunosignal remained in sgCHMP6 cells (Fig. 3*A*, *left graph*; *bottom panel*). Consistent with their detection in genetic screening, both sgVPS37A and sgCHMP6 cells displayed increased abundance of SLC20A1 protein compared with sgControl cells (Fig. 3*A*, *left graph*; *top panel*). In order to assess if increased SLC20A1 protein abundance was a result of increased *SLC20A1* mRNA abundance, we quantified *SLC20A1* mRNA in sgVPS37A-HEK293T and sgCHMP6-HEK293T cells with sgControls (Fig. 3*A*, *right graph*). *SLC20A1* mRNA levels were comparable between groups.

### Inhibition of protein degradation pathways reveals a role of lysosomal function in SLC20A1 protein degradation

Following ESCRT-dependent sorting of proteins in late endosomes, the final step of ESCRT-dependent protein degradation occurs in lysosomes. Pharmacological disruption of the lysosomal protein degradation pathway using bafilomycin A1 resulted in increased SLC20A1 protein levels (Fig. 3*B*, *top*), which is consistent with our genetic loss-of-function data. In contrast, disruption of the proteasomal protein degradation pathway using the proteasome inhibitor MG-132 did not result in comparable increases of SLC20A1 protein levels (Fig. 3*B*, *top*). As both lysosomal and proteasomal protein degradation pathways require substrate ubiquitination prior to degradation, inhibition of either pathway is known to result in accumulation of ubiquitinated proteins (23). Accordingly, we observed increased amounts of ubiquitinated proteins following treatment with either bafilomycin A1 or MG-132, confirming that both treatments were effective (Fig. 3*B*, *bottom*).

### SLC20A1 colocalizes with dominant-negative CHMP6-enhanced GFP

To examine if SLC20A1 is spatially associated with the ESCRT complex, we tested expression of CHMP6-enhanced GFP (EGFP) in cultured cells, which leads to the accumulation of degradation-bound proteins that overlap with CHMP6-EGFP (24). CHMP6-EGFP-transfected cells were immunostained for SLC20A1 with Alexa 594–based fluorescent secondary antibody, and confocal images revealed that some cells contained intracellular SLC20A1 punctae and compartments. A subset of these compartments colocalized with CHMP6-EGFP (SLC20A1 and CHMP6 panel of Fig. 3*C*, *white arrow*s), which places SLC20A1 in the same intracellular compartment as ESCRT machinery.

### *VPS37A*- and *CHMP6*-deficient cells display increased phosphate uptake

In order to probe if and how loss of ESCRT proteins affects cellular phosphate homeostasis in addition to SLC20A1 protein abundance, we measured actual phosphate uptake in sgVPS37A and sgCHMP6 HEK293T cells compared with sgControl cells. As expected, phosphate uptake was low in the absence of Na^+^ ions in incubation medium regardless of genotype, as most phosphate is transported through Na^+^/phosphate symporters including SLC20A1 (Fig. 3*D*). Accordingly, utilization of Na^+^-containing incubation medium resulted in increased phosphate uptake in sgControl cells. Na^+^-dependent phosphate uptake was significantly further elevated in both sgVPS37A and sgCHMP6 cells (Fig. 3*D*), consistent with the elevated SLC20A1 protein levels observed in our ESCRT perturbation experiments (Fig. 3*A*).

## Discussion

While phosphate is an essential metabolite for cells and perturbations in cellular phosphate levels contribute to myriad metabolic diseases (1, 2, 3, 4, 5, 7), it is rather surprising that the mechanisms underlying the regulation of phosphate homeostasis in mammalian cells remain poorly characterized. With the dual driving force of a 10-fold inward Na^+^ concentration and a negative interior voltage, the electrogenic SLC20A1 cotransporter will certainly overload the cells with calamitous amounts of phosphate. Thus, it is critical for the cell to limit cell surface SLC20A1 protein, gate its activity down, or both, for cell survival. Using a global CRISPR-based screening approach, we show that the ESCRT pathway tonically controls the cellular abundance of phosphate transporter SLC20A1, that SLC20A1 protein abundance is regulated principally at the post-transcriptional level, and that SLC20A1 is targeted for endolysosomal degradation by the canonical ESCRT machinery, thus matching cellular phosphate uptake to changes in phosphate requirement. Additional screening results unveil numerous novel candidate regulators of mammalian phosphate homeostasis including many subunits of the respiratory chain and oxidative phosphorylation pathways and several regulators of protein ubiquitination. This CRISPR-based whole-genome screening approach with a physiologic readout is a very powerful tool to address complex questions such as the regulatory mechanisms governing SLC20A1 abundance and activity when there is no clear candidate-based portal of entry for the interrogation.

**Figure 4:**
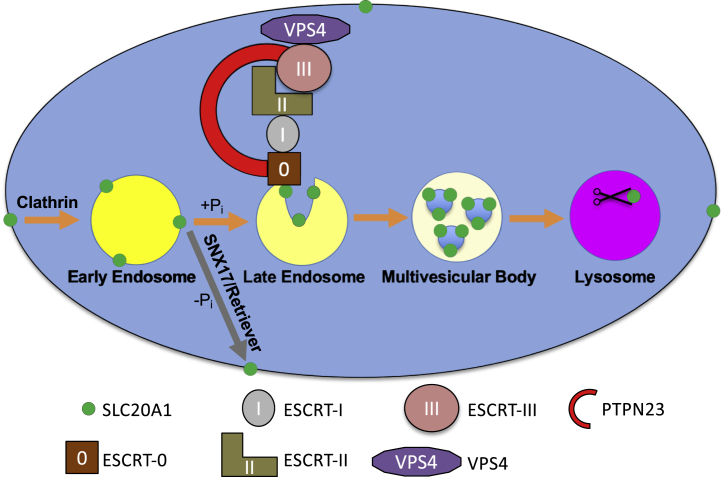
**Model of ESCRT complex–mediated negative regulation of SLC20A1 protein levels through degradation.** −Pi denotes phosphate-starved and +Pi phosphate-replete conditions. SLC20A1 is removed from the plasma membrane through clathrin-mediated endocytosis and incorporated into early endosomes. Under +Pi conditions, SLC20A1-containing early endosomes mature to late endosomes, and during this process, ESCRT-0, ESCRT-I, ESCRT-II, and accessory subunit PTPN23 are recruited. These factors act as scaffold for recruitment of ESCRT-III and VPS4, which mediates formation of SLC20A1-containing multivesicular bodies that are ultimately degraded in lysosomes. In addition, we propose that SLC20A1 is recycled from early endosomes back to the plasma membrane using the retriever complex and its cargo adapter SNX17 under −P_i_ conditions. See text for additional details. ESCRT, endosomal sorting complexes required for transport.

ESCRT machinery is involved in various cellular processes, including autophagy, membrane repair, cytokinetic abscission, and the destruction of plasma membrane proteins through lysosomal degradation (25). For the latter, degradation-bound proteins first undergo endocytosis. This process typically depends on formation of clathrin-coated vesicles from the plasma membrane, and the presence of clathrin heavy chain 1 (*CLTC*) (26) and adaptor-related protein complex 2 subunit mu 1 (*AP2M1*) (27) among the screening hits (Table S2) support the notion that SLC20A1 internalization indeed depends on clathrin-mediated endocytosis (Fig. 4). Subsequently, internalized proteins bind to ESCRT-0, which leads to recruitment of ESCRT-I and ESCRT-II subunits (28, 29, 30, 31). ESCRT-I and II and the auxiliary subunit PTPN23 act in parallel as scaffolds for recruitment of ESCRT-III subunits (32, 33), and the resulting assembly mediates recruitment of VPS4 and formation of cargo-containing intraendosomal multivesicular bodies that are degraded in lysosomes (34, 35). Lysosomal protein degradation generally depends on acidification of the lysosomal lumen by V-type ATPase (ATP6V) (36), and the presence of various subunits among our screening hits indicates that this is also the case for SLC20A1 (Table S2). Our results demonstrate that the ESCRT–lysosomal axis is an important negative regulator of SLC20A1 protein abundance as ESCRT genes were highly enriched among the 0.5% brightest SLC20A1-stained cells. In support of our screening results, VPS37A is thought to be the main VPS37 isoform for endosomal protein sorting together with the accessory ESCRT-I subunit UBAP1 (37, 38), which was also significantly enriched in our screen in contrast to the two other known accessory subunits *MVB12A* and *MVB12B*. Interestingly, VPS37A is the only VPS37 isoform with an identified ubiquitin-binding UVE domain (39), suggesting that interaction with a ubiquitination site is important for SLC20A1 degradation. Our findings highlight the power of CRISPR/Cas9-based whole-genome genetic screening technology with optimized guide design (40) as a means to identifying entire pathways including relevant isoforms involved in a biologic process.

In our validation experiments, we were able to obtain sgVPS37A clones with complete VPS37A loss of function while the most efficient sgCHMP6 clone still expressed some CHMP6 protein. While this discrepancy may reflect random chance introduced by clonal selection, it is possible that some function of the ESCRT machinery that is essential for cell survival can be maintained in the absence of VPS37A through alternative mechanisms, for example, recruitment of one of the alternative VPS37 subunits and/or bypass of ESCRT I and II through the auxiliary subunit PTPN23 (33), whereas chronic complete loss of CHMP6 renders cells nonviable because of the lack of redundancy or alternative pathways. In support of this notion, *VPS37C* showed a trend toward enrichment in the brightest cells of our screen, making it a possible *VPS37A* substitute.

In addition to the role of the ESCRT–lysosomal axis for SLC20A1 protein degradation that we identified, we also found increased SLC20A1 protein abundance after administration of the proteasome inhibitor MG-132, albeit to a lesser extent. This finding indicates that SLC20A1 can alternatively be degraded through the proteasomal machinery. This is not surprising given the well-established roles of the proteasome for quality and quantity control of newly synthesized protein (41, 42). In line with this concept, a similar pattern has been reported for the plasma membrane potassium channel Kir2.1 (43). While our results suggest that both the proteasome and the ESCRT–lysosomal axis can mediate SLC20A1 protein degradation, the increased SLC20A1 protein levels following lysosomal inhibition compared with proteasomal inhibition and the absence of enrichment of proteasomal proteins among the top genes and pathways of our screen suggest that the ESCRT–lysosomal axis is the principal pathway for SLC20A1 protein degradation that cannot be substituted completely by the proteasome.

Our loss-of-function data suggest that SLC20A1 is a direct target for ESCRT-dependent turnover; however, an alternative possibility is that a positive regulator of SLC20A1 is degraded through ESCRT, and deletion of ESCRT machinery derepresses such a regulator. CHMP6-EGFP expression results in dominant-negative perturbation of ESCRT function with accumulation of degradation-bound proteins at the ESCRT complex and colocalization with CHMP6-EGFP (24). The observed colocalization of SLC20A1 and CHMP6-EGFP places SLC20A1 in the same intracellular compartment as the ESCRT machinery and is consistent with a defect in SLC20A1 lysosome delivery, therefore supporting that SLC20A1 is a direct ESCRT target.

While we find increased SLC20A1 protein levels following loss of multiple ESCRT subunits in our genetic screen, a limitation of this screening approach is that it is based on an antibody that binds to the large intracellular domain of SLC20A1, which requires permeabilization of the examined cells, and therefore does not allow us to determine if SLC20A1 was increased at the plasma membrane. However, the increased phosphate uptake in sgVPS37A and sgCHMP6 cells supports the notion that SLC20A1 is indeed increased at the plasma membrane of ESCRT-deficient cells and imply the presence of an active mechanism for routing SLC20A1 from endosomes back to the plasma membrane. Two such mechanisms have been reported: first the retromer complex that depends on the cargo adapter SNX27 for recycling of endosomal proteins back to the plasma membrane (44) and second the retriever complex that depends on the cargo adapter SNX17 (45). Interestingly, interrogation of changes in the cell surface proteome of cells lacking SNX17 revealed decreased SLC20A1 cell surface protein levels, and SLC20A1 contains an intracellular NxxY motif that is required for SNX17 recognition (45). While these data suggest involvement of SNX17/retriever in recycling of SLC20A1 from endosomes back to the plasma membrane (Fig. 4), further studies are needed to formally examine its involvement.

Our findings further suggest that decreased ESCRT-dependent SLC20A1 degradation in the setting of phosphate starvation is a candidate mechanism for the observed post-transcriptional induction of SLC20A1 protein. An important caveat is that the induction of SLC20A1 effected by knocking down *VPS37A* or *CHMP6* is less pronounced than with phosphate starvation. The observed discrepancy may be explained by remaining ESCRT function in sgVPS37A and sgCHMP6 cells because of VPS37A bypass and incomplete CHMP6 loss of function, respectively, or by upregulation of proteasome-dependent SLC20A1 degradation during loss of the ESCRT–lysosomal axis, as discussed previously. In addition, it is possible that additional or alternative pathways are induced and operative during phosphate deficiency. Future studies are needed to dissect the underlying mechanisms in detail.

In closing, while many of our top screening hits are related to ESCRT-related protein degradation, we have identified various additional significantly enriched genes (Table S2) and pathways (Fig. 2*D*) among the top hits of our screen without an established role in mammalian phosphate homeostasis. Our screen revealed several regulators of protein ubiquitination. As ubiquitination of plasma membrane proteins commonly routes them for degradation through the ESCRT–lysosomal axis (46), it is an attractive hypothesis that these genes may modify SLC20A1 protein in order to route it toward ESCRT-mediated degradation. This would seem more likely than modifications of ESCRT machinery itself since many other plasma membrane proteins are degraded through the ESCRT–lysosomal axis. Further studies are needed to dissect the mechanisms directing SLC20A1 interactions with ESCRT, and the candidate regulators identified in our screen provide a foundation for these next experiments. Regulated pathways also include multiple subunits of respiratory chain, oxidative phosphorylation pathways, and the Krebs cycle (denoted in pathway analysis as tricarboxylic acid cycle). Interestingly, several genes that are part of the oxidative phosphorylation machinery and the Krebs cycle were also identified as phosphate regulated in an RNAi-based genome-wide genetic screen in insect cells that used abrogation of high phosphate-induced extracellular signal–regulated kinase phosphorylation as readout (47). While it is noteworthy that oxidative phosphorylation and the Krebs cycle have thus been implicated as regulators of phosphate homeostasis in two metazoan species using different experimental strategies, it is unclear how these pathways may be mechanistically linked to phosphate homeostasis. These and additional data contained in our screen will serve as a rich resource for further studies into the mechanisms regulating mammalian phosphate homeostasis.

## Experimental procedures

### Cell culture

HEK293T (American Type Culture Collection [ATCC] CRL-3216), U-2 OS (ATCC HTB-96), HCT116 (ATCC CCL-247), and U-87 MG (ATCC HTB-14) cell lines were grown using separate solutions to avoid contamination with other cell lines. Given these safeguards, the utilized cell lines were not authenticated. Cells were cultured in Dulbecco's modified Eagle's medium (DMEM) (Gibco; catalog no.: 11965) with 10% heat-inactivated fetal bovine serum (VWR; catalog no.: 89510) and 1% penicillin/streptomycin (Gibco; catalog no.: 15140) in 95% O_2_/5% CO_2_ at 37 °C. For phosphate-starvation experiments, cells were washed once with 0.9% sodium chloride solution and treated for 6 - 48 h with phosphate-free DMEM (Gibco; catalog no.: 11971) with 10% dialyzed fetal bovine serum (Gibco; catalog no.: A3382001), 1% penicillin/streptomycin (Gibco; catalog no.: 15140), and in addition 1 mM NaCl (pH = 6.4) for the experimental group or 1 mM Na_2_HPO_4_/NaH_2_PO_4_ (pH = 6.4) for the control group. For experiments requiring repeated media exchanges, culture plates were pretreated for better adhesion with 5 mg/50 ml poly-d-lysine hydrobromide (Sigma–Aldrich; catalog no.: P6407) in cell culture–grade water, washed once in water, air-dried for 2 h, and stored at 4 °C until the day of the experiment. For lysosome and proteasome inhibition experiments, HEK293T cells were incubated for 24 h with 100 nM bafilomycin A1 (Tocris; catalog no.: 1334) or 1 μM MG-132 (Sigma-Aldrich; catalog no.: M7449) in dimethyl sulfoxide (DMSO); DMSO was used as control (Sigma-Aldrich; catalog no.: D8418). Before genetic screening, mycoplasma contamination was ruled out using universal mycoplasma detection kit (ATCC; catalog no.: 30-1012K).

### siRNA-mediated gene knockdown

Human *SLC20A1* was knocked down in HEK293T cells using manufacturer-validated Silencer Select siRNA (s13087) with *GAPDH* (4390849) as positive control and scrambled siRNA (4390843) as negative control (all Invitrogen). Cells were seeded in DMEM (Gibco) with 10% heat-inactivated fetal bovine serum (VWR), transfected the following day with 30 nM siRNA and Lipofectamine RNAiMAX transfection reagent (Invitrogen; catalog no.: 13778) for 24 h, and subsequently phosphate starved for 48 h before they were harvested.

### CRISPR/Cas9-mediated gene targeting

For genetic loss-of-function studies, a guide targeting *VPS37A* (GCATAAGGAGACATCCCACTT), a guide targeting *CHMP6* (CCAGATCGAAATGAAAGTGA), a guide targeting *SLC20A1* (GACATGAAACCAGACAACAG), and a nontargeting control guide (ACGGAGGCTAAGCGTCGCAA) (40, 48) were each cloned into LentiCRISPR v2 lentivirus, which was a gift from Feng Zhang (Addgene; catalog no.: 52961). For virus generation, HEK293T cells were cotransfected with guide-expressing LentiCRISPR v2 plasmid, pMD2.G envelope–expressing plasmid (Addgene; catalog no.: 12259; gift from Didier Trono), and psPAX2 packaging plasmid (Addgene; catalog no.: 12260; gift from Didier Trono) using FugeneHD transfection reagent (Promega; catalog no.: E2311). Subsequently, HEK293T cells were transduced with sgControl-lentivirus, sgVPS37A-lentivirus, sgCHMP6-lentivirus, or sgSLC20A1-lentivirus and 0.8 μg/ml polybrene (EMD Millipore; catalog no.: TR-1003-G) for more efficient virus uptake, positive clones were selected using 1 μg/ml puromycin (Sigma–Aldrich; catalog no.: P8833) for 3 to 5 days, and single clones were isolated and characterized.

### Transduction of genetic screening library

HEK293T cells were transduced in duplicate with lentiviral preparation of LentiCRISPR v2-based pooled human CRISPR-KO library (Brunello), which was a gift from David Root and John Doench (Addgene; catalog no.: 73179-LVC) (40). To that end, 3 × 10^5^ HEK293T cells were plated in each well of 20 poly-d-lysine–covered 12-well plates and transduced the following day with the addition of 0.8 μg/ml polybrene (EMD Millipore; catalog no.: TR-1003-G) to achieve a multiplicity of infection of <0.4 and coverage of >400×. About 24 h later, cells were trypsinized and split into cell culture dishes with 1 μg/ml puromycin (Sigma–Aldrich; catalog no.: P8833) for 7 days followed by 5 to 6 days of recovery from puromycin. During this time, cells were trypsinized and split whenever they approached subconfluency.

### Flow cytometry and cell sorting

For the flow cytometry experiment, cells were washed once in PBS, detached with Accutase (Sigma–Aldrich; catalog no.: A6964) for 7 min, diluted in DMEM with 10% fetal bovine serum to neutralize Accutase, strained through a 40 μm filter, centrifuged at 300*g* for 3 min, resuspended in PBS, and counted. About 2 × 10^6^ cells per condition were fixed, permeabilized, and stained using an intracellular staining flow assay kit (Novus; catalog no.: NBP2-29450) with primary antibody against SLC20A1 (Cell Signaling; catalog no.: 12765) and Alexa Fluor 488–conjugated secondary antibody (Cell Signaling; catalog no.: 4412). Cell permeabilization for flow cytometry, cell sorting, and immunofluorescence was necessary as the utilized primary antibody binds to the large intracellular loop of SLC20A1 in the vicinity of amino acid 290 per manufacturer documentation. For the cell sorting experiment, an aliquot of transduced HEK293T cells (8.1–9 × 10^7^ cells) was frozen at −80 °C for determination of the guide distribution. The remaining cells were stained as outlined previously, ca. 10^8^ cells were sorted, and the 0.5% brightest cells were isolated for DNA extraction. Flow cytometry and cell sorting were performed at the UT Southwestern Flow Cytometry Core Facility using a FACSCalibur flow cytometer (BD Biosciences) and MoFlo cell sorter (Beckman Coulter), respectively.

### Genomic DNA extraction

Genomic DNA from nonsorted cells was extracted using Masterpure complete DNA and RNA Purification kit (Lucigen; catalog no.: MC85200) with the addition of 20 ng/μl glycogen (Roche; catalog no.: 10901393001) during isopropranol (Fisher Scientific; catalog no.: A451SK-4) precipitation or using quick-DNA Midiprep plus kit (ZymoResearch; catalog no.: D4075) per manufacturer instructions. DNA was extracted from ≥3.6 × 10^7^ cells to achieve >300× library coverage. Genomic DNA from sorted cells was extracted using a previously published method (49) that was adapted to optimize DNA yield from formalin-fixed cells. Cell pellets were resuspended in 460 μl of 10 mM Tris–HCl and 1 mM EDTA with pH = 8, 10 μl 0.5 M EDTA, 20 μl 5 M NaCl, 10 μl 20% SDS, and 5 μl 20 mg/ml proteinase K (Qiagen; catalog no.: 19131) and then incubated in a thermomixer (Eppendorf) at 65 °C and 1000 RPM overnight. The following day, samples were cooled to room temperature, 10 μl 100 mg/ml RNAse A (Qiagen; 1007865) was added, and samples were incubated in a thermomixer at 37 °C for 1 h. Samples were washed twice with 500 μl fresh phenol/chloroform/isoamyl alcohol (25:24:1 v/v; Invitrogen; catalog no.: 15593031) and once in 500 μl chloroform (Fisher; catalog no.: C298-4). At each wash step, samples were vortexed for 15 s, centrifuged for 1 min at 18,000*g* at room temperature, and the upper phase was transferred to a new tube. About 1 ml ice-cold fresh 100% ethanol (Pharmco-Aaper; catalog no.: 111000200) and 1 μl 20 mg/ml glycogen (Roche; catalog no.: 10901393001) were added to the final top phase, samples were vortexed for 15 s, frozen at −20 °C for 1 h, and centrifuged for 10 min at 15,000*g* at 4 °C. The resulting pellets were washed in ice-cold fresh 70% ethanol, air-dried for 5 min, and resuspended in 30 μl H_2_O. Qubit dsDNA BR assay kit (Invitrogen) was used for DNA quantification.

### Library preparation for sequencing

PCR of genomic DNA was performed using ExTaq polymerase (Clontech; catalog no.: RR001A) to add adapters and barcoding primers for Illumina sequencing per instructions from Addgene and the Broad Institute (primer sequences in Table S3). PCR of unsorted reference cells was performed in 100 μl aliquots with amounts sufficient to ensure >300× library coverage. Each reaction contained 10 μl 10× reaction buffer, 8 μl dNTP, 0.5 μl 100 μl P5 primer mix, 1.5 μl ExTaq polymerase, 10 μl 5 μM P7 primer, 2 μg DNA, and H_2_O. Samples were heated in a Veriti thermal cycler (Applied Biosystems) to 95 °C for 1 min, cycled 24× (95 °C × 30 s, 53 °C × 30 s, and 72 °C × 30 s), followed by a final extension period at 72 °C × 10 min. PCR of the sorted 0.5% brightest cells was performed in one scaled-down 20 μl reaction. PCR product was purified using AMPure XP PCR purification kit (Beckman Coulter; catalog no.: A63880) using 180 μl beads per 100 μl PCR product and subjected to quality assessment using electrophoresis on a Tapestation 4200 system and real-time PCR on a Step One Plus instrument (both from Applied Biosystems).

### Next-generation sequencing and data analysis

Samples were sequenced at the UT Southwestern McDermott Center Next-Generation Sequencing Core on Illumina NextSeq 500 with read configuration as 76 bp single end and analyzed at the Bioinformatics laboratory. The fastq files were subjected to quality check using fastqc (version 0.11.5; http://www.bioinformatics.babraham.ac.uk/projects/fastqc) and fastq_screen (version 0.11.4; http://www.bioinformatics.babraham.ac.uk/projects/fastq_screen), and adapters were trimmed using an in-house script. The reference sgRNA sequences for human CRISPR Brunello lentiviral pooled libraries were downloaded from Addgene (https://www.addgene.org/pooled-library/). The trimmed fastq files were mapped to reference sgRNA library with mismatch option as “0” using MAGeCK (model-based analysis of genome-wide CRISPR/Cas9 KO) (50). Read counts for each sgRNA were generated, and median normalization was performed to adjust for library sizes of different samples. Positively and negatively selected sgRNAs between comparisons and genes were identified using the default parameters of MAGeCK. Genes with *p* values less than 0.05 were selected for further functional over-representation analysis using the WebGestalt tool with top 20 categories ranked by significance levels (51). The pathway gene sets from biological processes, cell components, and molecular functions of Gene Ontology (http://www.geneontology.org/), molecular pathways of Reactome (http://www.reactome.org/), and Kyoto Encyclopedia of Genes and Genomes (https://www.genome.jp/kegg/) were used to identify the enriched affected categories. Complete screening results can be found in Tables S1 and S2 grouped by guide RNA and gene, respectively.

### RNA analyses

Total RNA was extracted from cultured cells using RNeasy plus mini kit (Qiagen; catalog no.: 74134) per manufacturer instructions. RNA quality and concentration were assessed using Nanodrop spectrophotometer (Thermo Fisher Scientific). Any remaining DNA was digested by DNAse I (Roche; catalog no.: 04716728001) treatment, and complementary DNA was generated using high-capacity complementary DNA reverse transcription kit (Life Technologies; catalog no.: 43-688-13). Subsequently, real-time quantitative PCR was performed using SybrGreenER qPCR SuperMix (Invitrogen; catalog no.: 11760500) in a QuantStudio 7 Flex PCR System (Life Technologies) as reported previously (52) using primers for human *SLC20A1* and *36B4* mRNA (primer sequences in Table S3).

### Protein analyses

For Western blots, 10 to 15 μg total protein lysate were loaded per lane. Samples from 48 h phosphate-starvation experiments except the experiment involving U-87 MG cells were heated to 95 °C for 3 min, and samples for other Western blots were heated to 37 °C for 30 to 120 min. ECL (Pierce; catalog no.: 32209) or SuperSignal West Femto (Thermo Scientific; catalog no.: 34095) Western blotting substrate was used to develop Western blots, and ImageQuant LAS4000 luminescent imager (General Electric) or ChemiDoc MP imaging system (Bio-Rad) was used for image acquisition. For densitometry analyses, mean *gray* values of Western blot images were measured, and background was subtracted for both SLC20A1 and loading control using the ImageJ software (National Institutes of Health)–based Fiji platform (53). Densitometry values were normalized to the respective control group. Antibodies against SLC20A1 (Cell Signaling; catalog no.: 12765), GAPDH (Cell Signaling; catalog no.: 5174), VPS37A (Thermo Fisher Scientific; catalog no.: PA5-51161), CHMP6 (Abcam; catalog no.: ab235050), and ubiquitin (Cell Signaling; catalog no.: 43124) were used with rabbit immunoglobulin G–horseradish peroxidase secondary antibody (Cell Signaling; catalog no.: 7074). Antibody against β-actin (Abcam; catalog no.: ab49900) was horseradish peroxidase conjugated.

### Phosphate uptake

sgVPS37A-HEK293T, sgCHMP6-HEK293T, and sgControl-HEK293T cells were grown to confluency in 12-well plates, and ^32^P uptake was measured (54). Cells were rinsed in Na^+^-free solution and incubated for 5 min at 37 °C in ^32^P uptake solution consisting of 140 mM NaCl, 5 mM KCl, 1 mM MgCl_2_, 10 mM Hepes (pH 7.4), 0.1 mM KH_2_PO_4_, and 1 μCi/ml ^32^P (PerkinElmer; catalog no.: NEX053001MC). Na^+^-free controls contained 140 mM tetramethyl ammonium chloride (Sigma–Aldrich; catalog no.: T-3411) instead of NaCl. Uptake solution was removed, and cells were washed in ice-cold stop solution (140 mM NaCl, 1 mM MgCl_2_, and 10 ml Hepes, pH 7.4). Subsequently, cells were lysed in SDS lysis buffer (0.1% SDS in water) and subjected to liquid scintillation counting in an LS6500 scintillation counter (Beckman) and to DC protein assay kit II (Bio-Rad; catalog no.: 5000112).

### Immunofluorescence

For phosphate-starvation experiments, sgControl and sgSLC20A1-HEK293T cells were fixed for 18 min in 4% paraformaldehyde (Pierce; catalog no.: 28908) in PBS, washed 3× 5 min in PBS, incubated for 1 h in blocking and permeabilization solution containing 0.1% bovine serum albumin (Vector Laboratories; catalog no.: SP-5050), 5% goat serum (Jackson ImmunoResearch; catalog no.: 005-000-121), and 0.5% saponin (Sigma–Aldrich; catalog no.: S4521) in PBS, incubated in first antibody overnight (Cell Signaling; catalog no.: 12765), washed 3× 5 min in PBS, incubated in Alexa 488–conjugated secondary antibody (Cell Signaling; catalog no.: 4412) for 1 h at room temperature, washed 3× 5 min in PBS, and mounted using Prolong Gold with 4′,6-diamidino-2-phenylindole (DAPI) (Invitrogen; catalog no.: P36931). A very dim background signal was detectable in the Alexa 488 channel of sgSLC20A1 cells, which suggests a minor nonspecific interaction of the utilized SLC20A1 antibody. For CHMP6-EGFP transfection experiments, HEK293T cells were transiently transfected with CHMP6-EGFP (Addgene; catalog no.: 31806; gift from Daniel Gerlich) using FugeneHD transfection reagent (Promega; catalog no.: E2311), grown for 24 h, fixated after aspiration of incubation medium, permeabilized, blocked, and stained with SLC20A1 antibody (Cell Signaling; catalog no.: 12765) followed by Alexa 594–conjugated secondary antibody (Cell Signaling; catalog no.: 8889). Integrity of the CHMP6-EGFP construct was confirmed using Sanger sequencing (UT Southwestern McDermott Center Sanger Sequencing Core) and immunoblot for CHMP6 (Abcam; catalog no.: ab235050) and EGFP (Abcam; catalog no.: ab290) (data not shown). All images were acquired at the UT Southwestern O’Brien Kidney Research Core at room temperature using an Observer.Z1 microscope platform with Plan Apochromat 63×/1.4 Oil DIC M27 objective, LSM880 confocal microscope system with 405, 488, and 594 nm lasers, Zeiss LSM T-PMT detector, and Black Zen software system (all Zeiss). Images were processed using the ImageJ software–based Fiji platform (53). Brightness and contrast settings were identical across each experiment for all channels except DAPI in the phosphate-starvation experiment, which was set using the autoadjustment feature to adjust for changes in DAPI intensity between conditions.

### Statistical analyses

With the exception of next-generation sequencing results (see aforementioned), data were analyzed using GraphPad Prism 9 (GraphPad Software, Inc) using unpaired two-tailed *t* test, one-way ANOVA, or two-way ANOVA with Tukey’s multiple comparisons test, as appropriate. The level of significance was *p* < 0.05 in all cases. Data are reported as mean values ± SD.

## Data availability

All data are contained in the article.

## Dedication

This article is dedicated to the memory of Dr Jean Donald Wilson (1932–2021) in honor of his legacy as an inspiring scientist, wonderful colleague, and dear friend.

## Supporting information

This article contains supporting information.

## Conflict of interest

The authors declare that they have no conflicts of interest with the contents of this article.

## Figures and Tables

**Supplemental Figure S2:**