# Supplementary material for: Cellular abundance of sodium phosphate cotransporter SLC20A1/PiT1 and phosphate uptake are controlled post-transcriptionally by ESCRT
Source: J Biol Chem. 2022 Apr 18;298(6):101945. doi: 10.1016/j.jbc.2022.101945 (PMC9123275; doi:10.1016/j.jbc.2022.101945)
Supplement: Supplemental Figures S1 and S2 Legend [file mmc4.docx]

**Supporting Figure 1: SLC20A1 protein levels are increased after 6 hours in -P_i_  HEK293T cells.** HEK293T cells were phosphate-starved (-P_i_) with phosphate-replete controls (+P_i_). Immunoblot for SLC20A1 (top lane) and β-actin loading control (bottom lane) for cells that were treated with -P_i_ vs. +P_i_ media for 6 hours **(A)**, 12 hours **(B)**, and 24 hours **(C)**. SLC20A1//β-actin abundance was determined by densitometry and normalized to the +P_i_ group (top).

**Supporting Figure 2: Phosphate starvation also results in massive induction of SLC20A1 protein abundance in U-2 OS, HCT116, and U-87 MG cells.**  Cells were phosphate starved (-P_i_) for 48 hours with phosphate-replete controls (+P_i_). Immunoblot for SLC20A1 (top lane) and β-actin loading control (bottom lane) for U-2 OS **(A)**, HCT116 **(B)**, and U-87 MG cells **(C)**. SLC20A1//β-actin abundance was determined by densitometry and normalized to the +P_i_ group (top).
